# Etiological analysis of patients with sudden sensorineural hearing loss: a prospective case–control study

**DOI:** 10.1038/s41598-023-32085-7

**Published:** 2023-03-30

**Authors:** Wen Xie, Niki Karpeta, Busheng Tong, Jiali Liu, Haisen Peng, Chunhua Li, Sten Hellstrom, Yuehui Liu, Maoli Duan

**Affiliations:** 1 Department of Otolaryngology, Head and Neck Surgery, The Second Affiliated Hospital of Nanchang University, Nanchang, 330006 China; 2 Division of Ear, Nose and Throat Diseases, Department of Clinical Science, Intervention and Technology, Karolinska Institutet, Stockholm, Sweden; 3 Department of Otolaryngology Head and Neck and Audiology and Neurotology, Karolinska University Hospital, 171 76 Stockholm, Sweden; 4 Department of Otolaryngology, Head and Neck Surgery, The First Affiliated Hospital of Anhui Medical University, Hefei, China

**Keywords:** Neurological disorders, Risk factors

## Abstract

Sudden sensorineural hearing loss (SSNHL) is a multifactorial emergency disease. Until now, the etiology of SSNHL is still unknown. Previous studies regarding the etiology of SSNHL are clinical studies depending on clinical data collection and analysis. Due to the insufficient sample size or various selective bias in clinical studies, the results of these studies may be inaccurate. This prospective case–control study aimed at exploring the possible etiology and risk factors of SSNHL. We enrolled 255 SSNHL patients and 255 sex-, age- and residence-matched non-SSNHL subjects in the control group. Our study shows that there was no significant difference in the prevalence of comorbidities including hypertension and diabetes, as well as the incidence of smoking and drinking habits between the case and control groups (P > 0.05). In addition, the peripheral blood white blood cell count, neutrophil count, platelet-to-lymphocyte ratio (PLR) and fibrinogen level of the case group were significantly higher than those in the control group (P < 0.05). These findings suggest smoking, drinking, hypertension and diabetes may not be related to the onset of SSNHL. However, hypercoagulable state and inner ear vascular microthrombosis related to an elevated fibrinogen level might be the risk factors of the disease. In addition, inflammation play an important role of SSNHL onset.

**Trial Registration**: Chinese Clinical Trial Registry. Registration number: ChiCTR2100048991.

## Introduction

Sudden sensorineural hearing loss (SSNHL) is a multifactorial emergency disease. Until now, the etiology of SSNHL is still unknown, resulting in the lack of specific treatment targeting for its etiology. Previous studies reported that microarteriosclerosis, microthrombosis, infection and immune factors may be the pathogenesis of SSNHL^1,2^. Some risk factors and diseases which may directly or indirectly lead to the cochlear hair cells damage and the ultrastructural changes can result in SSNHL.

The risk factors associated to SSNHL include psychological factors and living habits, such as depression, smoking and drinking^3,4^. The impairment of auditory recognition function in patients with depression may be related to the occurrence of SSNHL^4^. Smoking and drinking may lead to inner ear arteriosclerosis and microthrombus formation, which will affect the inner ear blood supply and thus cause SSNHL. On the other hand, no such association was found by some previous reports. For example, Nakashima et al. found that smoking and drinking habits had no significantly increase the risk of SSNHL^5^, in particular smoking did not seem to be a causative factor of SSNHL^6,7^.

In addition to lifestyle, other comorbidities, such as hypertension, diabetes and hyperlipidemia may also be risk factors of SSNHL. These findings are supported by some case–control studies, which showed that SSNHL patients had a higher likelihood of these diseases than non-SSNHL controls^2,5,8–10^. These finding has been supported by a large sample size case–control study, including 514 patients with SSNHL and 2570 controls^11^. Moreover, SSNHL patients with these cardiovascular risk factors had a worse prognosis^2^. The pathogenesis of SSNHL caused by hypertension, diabetes and hyperlipidemia is that these diseases tend to cause internal auditory arteriosclerosis and microthrombosis, resulting in the disturbance of inner ear microcirculation and degeneration and necrosis of hair cells. In addition, these diseases may lead to ultrastructural changes, lipid deposition and metabolic abnormalities of inner ear hair cells. Although evidence showing these comorbidities as potential risk factors for SSNHL has been demonstrated in many studies, some studies show different results. For example, Berjis et al.^12^ found that compared with controls, SSNHL patients had a similar prevalence of hypertension and diabetes as healthy subjects. Rudack et al.^13^ found that hypercholesterolemia was not a major risk factor for SSNHL in their case control study. Until now, there is no study of a large number of cases to evaluate whether patients with hypertension, diabetes and hyperlipidemia do indeed have an increased incidence of SSNHL.

The test results of SSNHL patients also provide clues for the etiology of SSNHL. Many previous studies have shown that, compared with healthy individuals of control groups, some laboratory test results of SSNHL patients were abnormal. These laboratory findings include changes in the coagulation system (such as increased plasma levels of fibrinogen, antithrombin and factor VIII, and deficiency of protein C or protein S), parameters of hemorheology (increased blood and plasma viscosity, erythrocyte aggregation index and erythrocyte filtration index), biomarkers of vascular endothelial cell function (decreased brachial artery flow mediated dilation, expression of endothelial progenitor cells and circulating adhesion molecules), oxidative stress response (increased oxygen free radicals), homocysteine and folate levels, inflammatory indexes [such as leukocyte count, neutrophil to lymphocyte ratio (NLR) and platelet to lymphocyte ratio (PLR)] and autoimmune biomarkers (such as circulating immune complex, antinuclear antibody and complement^14–21^.

Although the possible etiology of SSNHL has been widely studied, its exact etiology is still unable to be confirmed. One obstacle for SSNHL etiological study is that it is difficulty to establish an animal model simulating SSNHL. Therefore, most studies of SSNHL are clinical studies depending on clinical data collection and analysis. Due to the insufficient sample size or various selective bias in clinical studies, the results of these studies may be inaccurate. In order to explore the possible etiology of SSNHL, we conducted a prospective case–control study, which included 255 SSNHL patients and 255 non-SSNHL patients matched by gender, age and residence. We investigate the possible etiology of SSNHL by comparing the comorbidity occurrence and laboratory test results of the SSNHL patients with control subjects.

## Materials and method

### Patients

This prospective study included 255 SSNHL patients and 255 controls consecutively hospitalized between June 2021 and December 2021 in the Second Affiliated Hospital of Nanchang University, a provincial tertiary referral hospital in southeast China. All the 255 SSNHL patients with complete medical record data were recruited in this study. The control groups were randomly selected from the non-SSNHL subjects consecutively hospitalized in our department at the same period. Each patient of the case group and control group was matched for age, sex, income, and the region where they lived.

### Inclusion criteria

Case group inclusion criteria were as follows: patients suffered from SSNHL and the diagnostic criteria were based on the latest guidelines revised by the American Academy of Otolaryngology-Head and Neck Surgery in 2019. That is, patients had abrupt sensorineural hearing loss of more than 30 dB in three contiguous frequencies within 72 hours^22^. The control group consisted of 255 non-SSNHL patients, including 84 patients with epiglottic cyst, 122 patients with vocal cord polyp, 44 patients with deviated nasal septum and 5 patients with nasal vestibular cysts.

### Exclusion criteria

Exclusion criteria of case group included following: patients with normal hearing or with hearing loss due to other causes, such as otitis media, Meniere's disease, otosclerosis, congenital deafness, presbycusis, vestibular schwannoma and inner ear malformation. Cases with insufficient medical data were also excluded.

The control group excluded criteria were as follows: patients with sudden sensorineural hearing impairment.

All patients in the case group and the control group combined with other diseases, which cause abnormal serum white blood cells, platelet counts and fibrinogen level, such as acute and chronic infectious diseases, bleeding and hematological diseases and malignant tumor.

The study was done in accordance with the ethical principles and approved by the Second Affiliated Hospital of Nanchang University Institutional Review Board. Written, informed consent was obtained from all patients and/or their guardians.

### Test procedure

**Figure 1:**
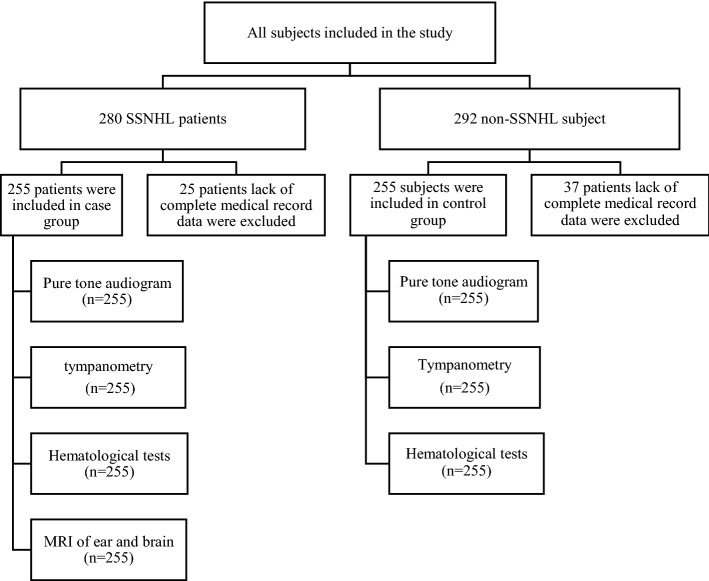
Flow diagram of the study.

All patients underwent detailed clinical interview. Clinical data, demographic information, past medical history and personal history were obtained. All patients underwent routine physical examination, general otorhinolaryngological examination, nervous system physical examination, audiological examination and laboratory tests. Magnetic resonance imaging (MRI) was conducted in all SSNHL patients. The flow gram of this study is shown in Fig. 1.

### Hearing evaluation

All patients’ hearing was evaluated with pure tone audiogram and tympanometry. All hearing tests were carried out by the same audiologist. Air and bone conduction were assessed at frequencies of 250 Hz, 500 Hz, 1 kHz, 2 kHz, 4 kHz, and 8 kHz. Pure tone average (PTA) was calculated as the mean of air conduction thresholds at 0.5, 1, 2, and 4 kHz^23^. The hearing loss levels were categorized into 5 grades: mild (26–40 dB HL), moderate (41–55 dB HL), moderate to severe (56–70 dB HL), severe (71–90 dB HL), and profound (> 90 dB HL)^24^. Audiogram patterns were classified into 5 types: ascending (the average threshold of 0.25–0.50 kHz was 20 dB higher than that of 4–8 kHz), descending (the average threshold of 4–8 kHz was 20 dB higher than that of 0.25–0.50 kHz), flat (all frequencies present similar thresholds and hearing threshold was below 80 dB HL), profound (all frequencies show similar threshold and hearing threshold was over 80 dB HL), and concave or convex type (average hearing degree of the mid-tone frequency was 20 dB higher than low and high frequencies)^25^.

### Hematological evaluation and comorbidities

Hematological tests were carried out in all patients. In addition to routine blood tests and biochemical tests, the parameters analyzed in this study were fasting blood sugar, hemostasis determinations, and lipid profile. The reference values which were defined normal in our laboratory are listed in Table 1.

**Table 1:** Normal reference values of blood test parameters.

Hematological tests	Hematological tests
Leukocyte count	3.5–9.5 × 10^9^/L
Neutrophil count	2–7 × 10^9^/L
Lymphocyte count	1.5–4 × 10^9^/L
Platelet count	125–350 × 10^9^/L
Fibrinogen	2–4 g/L
Total cholesterol	< 5.18 mmol/L
Triglycerides	< 1.7 mmol/L
High density lipoprotein cholesterol	Male: 1.16–1.42 mmol/L Female: 1.29–1.55 mmol/L
Low-density lipoprotein cholesterol	< 3.1 mmol/L

Comorbidities including hypertension and diabetes mellitus were also assessed. Hypertension was defined as blood pressure ≥ 140/90 mmHg^26^ or previous physician-diagnosed hypertension. DM was diagnosed according to the consensus of the expert committee on the diagnosis and classification of diabetes mellitus^27^, or diagnosed by internists and were treated with antidiabetic medications.

### Statistical analysis

Categorical data were shown as percentages and compared using Chi square test. Fisher exact test was used when expected counts in Chi square test were insufficient. Ranked data conforming to normal distribution and homogeneity of variance were assessed through Student’s T test, and Mann–Whitney 2-sample test was used for data which were not normally distributed. To evaluate the risk factors of SSNHL, odds ratio (or) and 95% confidence interval (95% CI) was calculated by univariate and multivariate logistic regression analysis. All analyses were conducted using SPSS version 25 for Windows. All statistical tests were 2-sided, and statistically significant levels were set at 0.05 (P < 0.05).

### Ethical approval

This study was approved by the Second Affiliated Hospital of Nanchang University Institutional Review Board. (reference number IIT-O-2021-002).

## Result

### Clinical characteristics, comorbidities and laboratory test results

**Figure 2:**
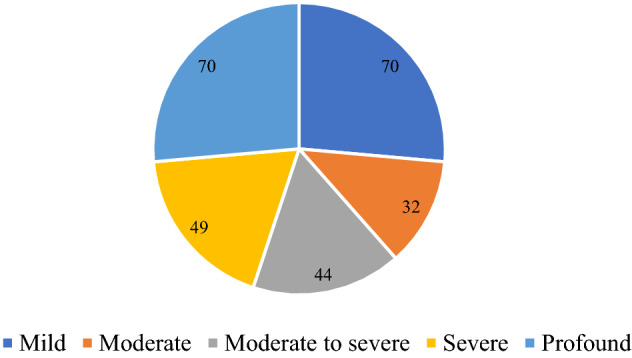
The numbers of affected ears in different hearing lever group.

**Figure 3:**
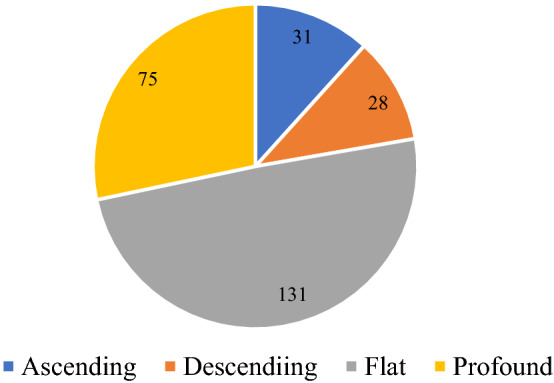
The numbers of affected ears in different audiogram type group.

**Figure 4:**
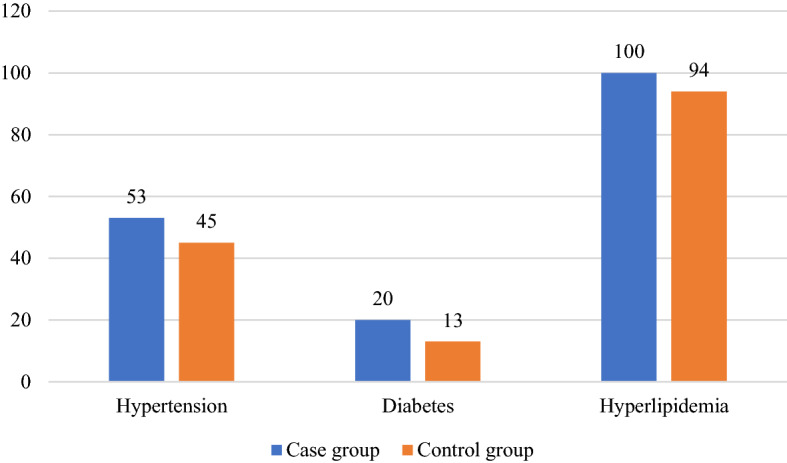
The numbers of subjects with comorbidities in the case and control groups.

Table 2 shows the clinical characteristics, comorbidities and laboratory test results of the two groups of patients. The average age of the patients in both groups was 46 years (range 12–83 years). Of the 255 patients in each group, 124 were males (48.6%) and 131 were females (51.4%). In the case group, SSNHL affected unilateral ear in 245 patients, and bilateral ears in 10 patients. Of the 245 unilateral ears, there were 116 affected left ears and 129 right ears. The duration from onset to treatment ranged from 1 to 30 days, with an average of 8.07 days. Regarding the degree of hearing loss in 265 ears of 255 patients, the most common one is profound (26.4%) and mild hearing loss (26.4%), followed by severe hearing loss (18.5%) and moderate to severe hearing loss (16.6%), moderate hearing loss was relatively rare (12.1%) (Fig. 2). Regarding audiogram shape, as depicted in Fig. 3, flat type hearing loss occurred in 131 ears (49.4%), pronounced hearing loss affected 75 ears (28.3%), followed by ascending (11.7%) and descending type (10.6%). The prevalence of comorbidity in the two groups was shown in Fig. 4. In the case group, 53 patients (20.8%) had comorbidities with hypertension, and 20 patients (7.8%) suffered from diabetes. In the control group there were 45 cases (17.6%) with hypertension and 13 cases with diabetes mellitus (5.1%). There was no significant difference in the incidence of comorbidity between the two groups (P > 0.05).

**Table 2:** Clinical characteristics, comorbidities and laboratory examination results of patients in case group and control group.

	Case group (n = 255)	control group(n = 255)	Statistical value	P value
Age (years), mean ± sd	46.47 ± 1.002			
Gender				
Male [cases (%)]	124 (48.6)			
Female [cases (%)]	131 (51.4)			
Comorbidities				
Hypertension [cases (%)]	53(20.8)	45(17.6)	1.706	0.425#
Diabetes [cases (%)]	20 (7.8)	13 (5.1)	1.588	0.28&
Hyperlipidemia [cases (%)]	100 (39.2)	94 (36.9)	0.299	0.584&
Personal history				
Smoking [cases (%)]	28 (11)	20 (7.8)	1.472	0.288&
Drinking [cases (%)]	21 (8.2)	13 (5.1)	2.017	0.156&
Laboratory test results				
Leukocyte count (× 10^9^/L), mean ± sd	6.98 ± 1.43	6.53 ± 0.13	-2.729	0.006@
Neutrophil count (× 10^9^/L), mean ± sd	4.667 ± 0.131	4,202 ± 0.128	-3.265	0.001@
Lymphocyte count (× 10^9^/L), mean ± sd	1.824 ± 0.455	1.807 ± 0.346	-0.281	0.779@
Platelet count (× 10^9^/L), mean ± sd	231.41 ± 6.469	224.5 ± 3.757	-0.064	0.946@
Ratio of neutrophils to lymphocytes, mean ± sd	1.87 ± 0.52	1.68 ± 0.51	-3.264	0.001@
Ratio of platelet lymphocyte, mean ± sd	147.74 ± 5.57	133.71 ± 3.16	-0.388	0.698@
Fibrinogen (g/L), mean ± sd	5.63 ± 1.61	4.6 ± 1.27	-5.728	0.000@
Cholesterol (mmol/L), mean ± sd	4.96 ± 0.07	4.91 ± 0.68	-0.538	0.59@
Triglyceride (mmol/L), mean ± sd	1.91 ± 0.12	1.85 ± 0.88	-1.719	0.086@
High density lipoprotein (mmol/L), mean ± sd	1.28 ± 0.03	1.27 ± 0.02	-0.088	0.93@
Low density lipoprotein (mmol/L), mean ± sd	2.86 ± 0.06	2.79 ± 0.05	-0.978	0.328@

^#^Fisher's exact test, ^&^The Chi-squared test, ^@^nonparametric Mann Whitney U test.

**Figure 5:**
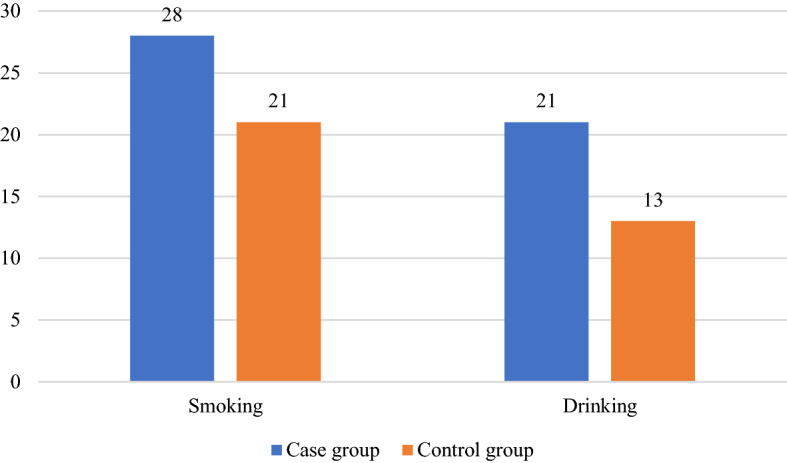
The numbers of subjects with smoking and drinking habits in the case and control groups.

The personal history of smoking and drinking habits in the two groups are shown in Fig. 5. In the case group, 28 patients (11%) had smoking habits and 21 patients (8.2%) had drinking habits. The percentage of subjects with smoking and drinking habits were 7.8% and 5.1%, respectively in the control group. There was no significant difference in the proportion of subjects with smoking and drinking history between the two groups (P > 0.05).

The laboratory examination results showed that there was no significant difference in peripheral blood lymphocyte count, platelet count, platelet-to-lymphocyte ratio (PLR), cholesterol, triglyceride, high-density lipoprotein and low-density lipoprotein levels of the patients between the two groups (P > 0.05). In contrast, the peripheral blood leukocyte count, neutrophil count, NLR and fibrinogen levels of the patients in case group were significantly higher than those in control group (P < 0.05).

### MRI result

All 255 SSNHL patients’ MRI of ear had normal results. Regarding brain MRI results, 137 patients’ results were normal, but old slight ischemia was visualized in 118 patients, and 4 of them had mild brain atrophy. The 4 latter patients were over 70 years old.

### Clinical characteristics, comorbidities, laboratory test and hearing results of SSNHL patients with different genders

We investigated the difference in terms of clinical characteristics, comorbidities, laboratory test and hearing results of male and female patients (Table 3). There was a significant difference in prevalence of hypertension and diabetes, proportions of patients with smoking and drinking habits, peripheral blood lymphocyte count, platelet count, PLR, fibrinogen, cholesterol, triglyceride and high-density lipoprotein between the male and female SSNHL patients (P < 0.05). Notably, the prevalence of hypertension in female patients was higher than in male patients, whereas the diabetes prevalence was lower in female patients. Additionally, almost all patients with smoking or drinking habits were males.

**Table 3:** Clinical characteristics, comorbidities prevalence, laboratory examination and hearing results of male and female SSNHL patients.

	Male (n = 124)	Female (n = 131)	Statistical value	P
Age (years), mean ± standard deviation	45.81 ± 15.684	47.09 ± 15.836		0.516^^^
Comorbidities				
Hypertension [cases (%)]	29(23.4)	24(18.3)	345.34	0.000^&^
Diabetes [cases (%)]	7 (5.6)	13 (9.9)	346.176	0.000^&^
Hyperlipidemia [cases (%)]	43 (34.7)	57 (43.5)	2.086	0.149^&^
Personal history				
Smoking [cases (%)]	27 (21.8)	1 (0.8)	382.811	0.000^&^
Drinking [cases (%)]	20 (16.1)	1 (0.8)	370.848	0.000^&^
Laboratory test results				
Leukocyte count (× 10^9^/L), mean ± standard deviation	7.037 ± 0.191	6.926 ± 0.212	− 0.865	0.387^@^
Neutrophil count (× 10^9^/L), mean ± standard deviation	4.584 ± 0.172	4,745 ± 0.195	− 0.217	0.829^@^
Lymphocyte count (× 10^9^/L), mean ± standard deviation	1.903 ± 0.063	1.748 ± 0.065	− 0.2056	0.04^@^
Platelet count (× 10^9^/L), mean ± standard deviation	228.48 ± 11.94	234.18 ± 5.601	− 2.671	0.008^@^
Ratio of neutrophils to lymphocytes, mean ± standard deviation	2.872 ± 0.194	3.312 ± 0.222	− 1.715	0.086^@^
Ratio of platelet lymphocyte, mean ± standard deviation	138.144 ± 9.107	156.822 ± 6.507	− 3.245	0.001^@^
Fibrinogen (g/L), mean ± standard deviation	5.486 ± 2.229	5.768 ± 2.326	− 2.991	0.003^@^
Cholesterol (mmol/L), mean ± standard deviation	4.800 ± 0.105	5.118 ± 0.098	− 2.295	0.022^@^
Triglyceride (mmol/L), mean ± standard deviation	2.197 ± 0.166	1.629 ± 0.181	− 4.011	0.000^@^
High density lipoprotein (mmol/L), mean ± standard deviation	1.145 ± 0.026	1.410 ± 0.041	− 6.104	0.000^@^
Low density lipoprotein (mmol/L), mean ± standard deviation	2.785 ± 0.088	2.937 ± 0.070	− 1.703	0.089^@^
Type of auditory curve				
Ascending type [cases (%)]	12 (9.7)	19 (14.5)	6.159	0.155^#^
Descending type [cases (%)]	17 (13.7)	10 (7.6)		
Flat type [cases (%)]	63 (50.8)	59 (45)		
Profound [cases (%)]	31 (25)	43 (32.8)		
Degree of hearing loss				
Mild [cases (%)]	31 (25)	38 (29)	1.884	0.757^&^
Moderate (%))	17 (13.7)	13 (9.9)		
Moderate to severe [cases (%)]	21 (16.9)	19 (14.5)		
Severe [cases (%)]	24 (19.4)	23 (17.6)		
Profound [cases (%)]	31 (25)	38 (29)		

^^^T test, ^&^The Chi-squared test, ^@^nonparametric Mann Whitney U test, ^#^Fisher's exact.

### Analysis of possible risk factors of SSNHL

Univariate and multivariate logistic regression analysis were used to evaluate the strength of association between hypothesized risk factors and the likelihood of SSNHL (Tables 4, 5). The results showed that hypertension, diabetes and hyperlipidemia, and smoking or drinking habits were not be associated with SSNHL.

**Table 4:** Univariate logistic regression analysis of possible risk factors for SSNHL.

	Unadjusted OR	95% CIs	P value
Hypertension			
No	Ref (1)		
Yes	1.192	0.768–1.851	0.434
Diabetes			
No	Ref (1)		
Yes	1.584	0.770–3.258	0.218
Hyperlipidemia			
No	Ref (1)		
Yes	0.905	0.633–1.294	0.584
Smoking			
No	Ref (1)		
Yes	1.449	0.794–2.646	0.227
Drinking			
No	Ref (1)		
Yes	1.671	0.818–3.414	0.159

**Table 5:** Multivariate logistic regression analysis of possible risk factors for SSNHL.

	Adjusted OR	95% CIs	P value
Hypertension			
No	Ref (1)		
Yes	1.060	0.669–1.678	0.805
Diabetes			
No	Ref (1)		
Yes	1.590	0.762–3.318	0.216
Hyperlipidemia			
No	Ref (1)		
Yes	0.925	0.644–1.328	0.672
Smoking			
No	Ref (1)		
Yes	1.152	0.485–2.736	0.749
Drinking			
No	Ref (1)		
Yes	1.481	0.533–4.110	0.451

## Discussion

The results of our prospective case–control study on sudden sensorineural hearing loss neither showed any significant relation to comorbidities by hypertension or diabetes or hyperlipidemia nor to living habits as smoking and drinking habits. This is in agreement with previous studies conducted by Nakashima et al.^5^ and Mosnier et al.^6^ but contrasts to other studies by Passamonti^28^ and Lin et al.^3^, of which the latter was a systematic review and meta-analysis study. Lin et al. reported that smoking was risk factor of SSNHL, but this finding was discordant with the result of systematic review and meta-analysis conducted by Saba et al.^29^. The size of the cohort and the population-based data set in various studies could account for the controversial results. Another reason of this discrepancy regarding our study is that there was low proportion of smoking and drinking patients in both the case and control groups in our study, which may result in no significant difference in statistical values although there is a somewhat higher incidence in the case group than control group.

Our study did not show that significant difference exists in the prevalence of hypertension, diabetes and hyperlipidemia between SSNHL patients and controls. Furthermore, the difference in OR value is not statistically significant between the patients of the two groups, suggesting that hypertension, diabetes and hyperlipidemia is not related to the onset of SSNHL. Our previous retrospective study also supports this conclusion, which found that the prevalence of hypertension and diabetes in SSNHL patients was not higher than in the local population^30^. Hyperlipidemia, as a risk factor of SSNHL, has been reported by two systematic review and Meta-Analysis^29,31^. Regarding the prevalence of hypertension and diabetes in SSNHL patients, he results of these two studies are inconsistent. Saba et al. found SSNHL patients had high risk of hypertension and diabetes^29^, but Simões et al. reached different conclusion after pooled analysis of adjusted ORs^31^. Our finding is also inconsistent with that of previous studies^2,5,8,9^ and except differences in sample size and population one possible explanation of this discordant result could be that the enrolled subjects in our control group were non-SSNHL patients rather than healthy people.

As mentioned before, vascular factors and thrombosis may be an important cause of SSNHL, and the increase of blood viscosity and hypercoagulable state of blood may contribute to microthrombosis. In agreement with previous studies^32,33^, our study showed that the serum fibrinogen level of SSNHL patients was higher than that of the controls. Moreover, another case–control study obtained similar results, but there was no significant difference in the levels of triglycerides, low-density lipoprotein and high-density lipoprotein between SSNHL patients and controls^13^. Although most studies have confirmed that hyperfibrinogenaemia may lead to SSNHL, previous works conducted by Passamonti et al. did not support this hypothesis^28^. His study enrolled 118 SSNHL patients and 415 controls and found that the levels of serum VIII and homocysteine increased, and antithrombin protein C decreased in SSNHL patients, but no significant difference existed in fibrinogen level between subjects in the two groups. In our study, we found that hyperfibrinogenaemia may be a risk factor for SSNHL, due to a mechanism that an elevated fibrinogen level may increase the risk of microthrombosis. As we all know, plasma viscosity mainly depends on the amount of fibrinogen, which is the most abundant plasma protein. It is an indicator of hypercoagulability and hypofibrinolysis, and is the premise for thrombosis. The fibrinogen content in SSNHL patients is higher than that in controls, suggesting that hypercoagulability is more common in SSNHL patients. This in line with the Chinese guidelines for the diagnosis and treatment of SSNHL revised in 2015 that pointed out that a disturbed inner ear embolism or thrombosis may be the pathogenesis of profound SSNHL^25^. Most SSNHL patients referred to our hospital which suffered from severe to profound hearing loss, also had an elevated fibrinogen level which supports this hypothesis.

In addition to regulate coagulation, fibrinogen is a product and potent driver of inflammation. On one hand, higher plasma fibrinogen can be caused by acute inflammation, in which inflammatory cytokines can stimulate increased hepatic expression of fibrinogen. On the other hand, fibrinogen are key functional factors in mediating inflammatory response. Firstly, fibrinogen can promote leukocyte to migrate out of the vascular system by acting as a bridging molecule to promote intercellular adhesion, as it is a ligand for many cell surface receptors expressed by leukocytes endothelial cells and other types of cells^34^. Secondly, fibrinogen can profoundly change leukocyte function by changing cell movement, phagocytosis, NF-B–mediated transcription, production of chemokines and cytokines degranulation^35–38^. Collectively, the elevated plasma fibrinogen among SSNHL patients indicate that SSNHL is both an inflammation-driven coagulation disease and coagulation-driven inflammation disease.

Another important finding in our study to confirm the role of inflammation in SSNHL is that the peripheral blood leukocyte count, neutrophil count and NLR of patients in the case group are higher than those of the control group. This is in accordance with other case–control studies. Thus, Seo et al.^18^ revealed that the NLR and PLR of SSNHL patients were higher than those of the control group. In addition, they found that the NLR of the unrecovered patients was significantly higher than the recovered patients. Therefore, they concluded that NLR was a reliable predictor for the prognosis of SSNHL. Similar results have also been reported by Ulu et al.^39^, Qiao et al.^40^ and Masuda et al.^41^. They found that the NLR and PLR of the case group were higher than those of the control group. Additionally, Ulu documented that the systemic immune inflammation index of the case group was also higher among SSNHL patients. The results of a meta-analysis also supported this finding and also showed that NLR is a prognostic biomarker of SSNHL^42^. It is widely accepted that that peripheral leukocyte count is an indicator of inflammatory reaction and elevated NLR reflects the active inflammatory activities as well. These findings suggest that inflammation is involved in the pathogenesis of SSNHL. Previous studies have also demonstrated that NLR is a marker of other peripheral vascular afflictions than atherosclerosis and arterial thrombosis, such as acute coronary syndrome, heart failure and end-stage renal disease. Some scholars believed that the increase of these inflammatory indicators will support the theory that systemic stress activates inflammatory reaction, resulting in endothelial function damage, which leads to atherosclerosis and ischemic events of inner ear. This process is characterized by the increase of NLR and PLR^18^. Patients with higher values of inflammatory indexes also had a poor prognosis, indicating that patients with severe endothelial cell damage had worse treatment effects^18,40,41^. This finding also confirms the effectiveness of glucocorticoids in SSNHL treatment by inhibiting inflammatory response, which is also unanimously supported by SSNHL treatment guidelines in various countries.

Our finding is in agreement with those of previous studies reporting that inner ear microcirculation disorder, arteriosclerosis, arterial thrombosis or embolism and immune factors may be the main causes of SSNHL. However, the etiology of most SSNHL patients is still unclear, further studies will be needed to explore the etiology of SSNHL.

### Strengths and limitations of this study

The primary strength of our study is that it is a prospective case–control study with a large sample size. In addition to analyzing the possible risk factors of SSNHL. Moreover, we explored the clinical characteristics, comorbidities, laboratory test results and imaging examination results of SSNHL patients. We investigated the difference in terms of clinical characteristics, comorbidities, laboratory test and hearing results of male and female patients. which can provide useful information for the readers in the field.

This study had some limitations. Firstly, the subjects enrolled in control group enrolled were non-SSNHL patients instead of healthy individuals, which may affect the accuracy of the study results. Secondly, there may existed some self-report bias regarding the personal history collection process, since participants may conceal their smoking or drinking habits due to people’s negative attitude towards these living habits in China. We found that the self-reported proportion of patients with smoking and drinking was very low both in case and control group.

## Conclusions

Our prospective case–control study on SSNHL did not demonstrate any comorbidities with hypertension, diabetes or hyperlipidemia. Neither could any relation to living habits like drinking and smoking be shown.

Interestingly our study demonstrated an increased level of serum fibrinogen among SSNHL patients which suggests that a hypercoagulable state and an inner ear vascular microthrombosis might be the risk factors of the disease. This is also supported by the fact that the elevated peripheral blood white blood cell count, neutrophil count, and NLR in SSNHL indicate that inflammation plays an important role of SSNHL onset.

## Data Availability

After publication of the primary and secondary analyses detailed in the individual deidentified patient data, including a data dictionary, will be made available via our data sharing website indefinitely (web site link: https://mrhuajian.github.io/index.html) or from the corresponding author on reasonable request.
